# Measuring Professional Competency in School Counselors: Development and Rasch Validation of a Multidimensional Instrument

**DOI:** 10.12688/f1000research.178940.1

**Published:** 2026-04-22

**Authors:** Deasy Yunika Khairun, Agus Taufiq, Yusi Riksa Yustiana, Fuja Siti Fujiawati, Maya Nurlita, Sohibun, Kamaliyah Kadir, Mutmainnah, Ibrahim Al Hakim

**Affiliations:** 1 Guidance and Counseling, Universitas Sultan Ageng Tirtayasa, Serang, Banten, 42111, Indonesia; 2 Guidance and Counseling, Universitas Pendidikan Indonesia, Department of Educational Psychology and Guidance, Bandung, West Java, 40150, Indonesia; 3 Guidance and Counseling, Universitas Pendidikan Indonesia, Department of Educational Psychology and Guidance, Bandung, West Java, 40150, Indonesia; 4 Educational Curriculum, Universitas Sultan Ageng Tirtayasa, Serang, Banten, 42111, Indonesia; 5 Mathematics Education, Universitas Dayanu Ikhsanuddin, Baubau, Southeast Sulawesi, 93716, Indonesia; 6 Physics Education, Universitas Pendidikan Indonesia, Bandung, West Java, 40150, Indonesia; 7 Mathematics Education, Indonesia University of Education Department of Mathematics Education, Bandung, West Java, 40150, Indonesia; 8 Guidance and Counseling, Sekolah Tinggi Keguruan dan Ilmu Pendidikan Muhammadiyah Enrekang, Enrekang, South Sulawesi, 91712, Indonesia; 9 Guidance and Counseling, Universitas Pendidikan Indonesia, Department of Educational Psychology and Guidance, Bandung, West Java, 40150, Indonesia

**Keywords:** Professional Competency, School Counselors, Rasch Measurement Model, Instrument Development, Construct Validity, Psychometric Validation

## Abstract

**Background:**

The measurement of professional competency in school counseling remains methodologically constrained by fragmented domain assessment and heavy reliance on classical test theory approaches. There is a critical need for an empirically calibrated instrument that integrates regulatory competency standards with expanded theoretical domains while ensuring measurement precision and structural coherence. This study developed and validated a multidimensional instrument of professional competency for school counselors using Rasch analysis.

**Methods:**

A cross-sectional instrumental design was implemented. The instrument was constructed across seven domains: assessment, development of guidance and counseling program, implementation of guidance and counseling services, evaluation of guidance and counseling program, supervision of pre-service counselor training, continuous professional development, and collaborative engagement in co-curricular programs. Seventy-one items were rated on a five-point Likert scale. Data from 93 practicing school counselors were analyzed using the Rasch Rating Scale Model. Analyses included the evaluation of category functioning, item and person fit statistics, reliability and separation indices, and principal component analysis of residuals (PCAR) to assess construct dimensionality.

**Results:**

The Rasch model demonstrated acceptable item fit and ordered rating scale thresholds. The raw variance explained by measures was 53.4%, with 6.7% unexplained variance in the first contrast, indicating a dominant latent construct despite the multidomain theoretical structure. Item difficulty estimates ranged from −1.68 to +1.71 logits, forming a hierarchical continuum from foundational service practices to advanced supervisory and professional roles. Reliability and separation indices indicated stable item calibration and adequate differentiation among levels of professional competency.

**Conclusions:**

The findings support the structural validity, hierarchical calibration, and measurement precision of the instrument within a unified Rasch framework. The instrument provides a methodologically robust basis for professional assessment, supervision, and competency-based development in school counseling contexts, with significant potential for further validation across diverse educational systems.

## Introduction

Professional competency is widely recognized as a foundational determinant of service quality and ethical practice in school counseling ( Kaslow, 2004; Remley & Herlihy, 2010). As educational systems worldwide increasingly emphasize accountability, evidence-based intervention, and student well-being, the role of school counselors has expanded beyond traditional guidance functions toward comprehensive developmental, preventive, and systemic responsibilities ( Yuwono & Utomo, 2023; Zabek et al., 2023). In this context, the measurement of professional competency becomes not merely evaluative but strategic informing supervision, professional development, certification, and policy implementation ( Borders, 2014; Stolenberg & McNeill, 2010).

The construct of professional competency in school counseling is inherently complex and theoretically multidimensional. Regulatory frameworks, such as national counseling standards and professional accreditation systems, emphasize core domains including needs assessment, guidance and counseling program planning, service implementation, and guidance and counseling program evaluation ( Permendikbud No. 111, 2014). In the Indonesian context, for instance, the Regulation of the Minister of Education and Culture No. 111/2014 formally articulates four central competencies: (1) assessment of student potential, characteristics, and environment, (2) development of counseling programs, (3) implementation of guidance and counseling services, and (4) evaluation of guidance and counseling program. These domains represent the operational foundation of professional practice.

However, contemporary scholarship argues that professional competency extends beyond technical-programmatic capabilities. The literature emphasizes the importance of supervisory competency, continuous professional self-development, ethical accountability, and systemic collaboration within the broader educational ecosystem ( Kivlighan, 2008; Luke & Bernard, 2006; Riggs & Bartholomaeus, 2016; Vivekananda et al., 2021). Notably, three dimensions frequently underrepresented in Western measurement models include: (a) the supervision of pre-service counselor training ( Littrell et al., 1976; Luke & Bernard, 2006), (b) sustained personal and professional growth ( Stoll et al., 2006; Young et al., 2011), and (c) active collaboration with school personnel in co-curricular and systemic initiatives ( Yudha et al., 2024; Zabek et al., 2023). These dimensions are critical for sustaining the profession, ensuring service quality, and integrating counselors into formal educational structures ( Kaslow, 2004; Rice & Furr, 2013). Their inclusion reflects a more holistic and sustainability-oriented conception of professional competency.

Despite the acknowledged multidimensionality of professional competency, existing measurement instruments reveal several limitations. First, many instruments are fragmented, assessing isolated domains rather than an integrated competency system ( Ridley et al., 2021). Second, numerous tools are grounded in Western-centric professional frameworks that may not fully capture the regulatory and systemic realities of school counseling in emerging educational contexts ( Khairun, Taufiq, Yustiana, Budiman, & Al Hakim, 2025a; Khairun, Taufiq, Yustiana, Budiman, & Hakim, 2025b). Third, classical test theory (CTT) approaches continue to dominate validation procedures, often limiting measurement precision and failing to provide invariant item calibration across varying respondent ability levels ( Sumintono & Widhiarso, 2015). Consequently, there remains a need for a psychometrically robust, multidimensional instrument that integrates regulatory foundations with contemporary theoretical advancements while employing modern measurement models.

The Rasch Measurement Model offers a rigorous alternative to traditional CTT-based validation ( Bond, 2015; Boone et al., 2013; Sumintono & Widhiarso, 2015). By transforming ordinal raw scores into interval-level measures, Rasch analysis enables objective calibration of item difficulty and respondent ability on a common metric ( Linacre, 2016; Sumintono & Widhiarso, 2015). Furthermore, Rasch-based principal component analysis of residuals provides empirical evidence of dimensional structure, ensuring that theoretically multidimensional frameworks cohere into a unified latent construct ( Boone et al., 2013; Zaporozhets et al., 2015). This approach enhances measurement precision, supports invariance, and strengthens interpretability in applied professional contexts.

Grounded in both regulatory mandates and international theoretical perspectives, the present study develops a multidimensional Professional Competency Instrument for School Counselors comprising seven integrated domains: assessment, development of guidance and counseling program, implementation of guidance and counseling services, evaluation of guidance and counseling program, supervision of pre-service counselor training, continuous professional development, and collaborative engagement in co-curricular programs ( Luke & Bernard, 2006; Permendikbud No. 111, 2014; Stoll et al., 2006). Although theoretically multidimensional, these domains are conceptualized as interrelated components of a single overarching construct professional competency in school counselors.

Although previous research has examined components of counselor competency and professional identity, existing instruments tend to assess fragmented skill sets or remain anchored in narrowly defined programmatic domains ( Khairun, Taufiq, Yustiana, Budiman, & Al Hakim, 2025a; Khairun, Taufiq, Yustiana, Budiman, & Hakim, 2025b; Ridley et al., 2021). Moreover, competency models frequently underrepresent supervisory engagement, sustained professional development, and systemic collaboration domains that are increasingly recognized as essential to the sustainability and institutional integration of school counseling practice ( Luke & Bernard, 2006; Stoll et al., 2006; Vivekananda et al., 2021). Methodologically, many prior validation studies rely predominantly on classical test theory, which does not ensure invariant measurement or hierarchical calibration of item difficulty across varying respondent ability levels ( Sumintono & Widhiarso, 2015). By integrating regulatory competency mandates with expanded theoretical domains into a unified seven-domain framework and validating the construct using the Rasch Measurement Model, the present study advances both the conceptual architecture and the psychometric rigor of professional competency measurement in school counselors. This dual advancement strengthens the coherence, precision, and applicability of competency assessment within contemporary educational systems.

Accordingly, this study aims to develop and rigorously validate a multidimensional instrument for measuring professional competency in school counselors using a five-point Likert rating scale and Rasch Measurement Model analysis. Specifically, the study seeks to (1) evaluate rating scale functioning across ordered response categories, (2) examine construct validity through principal component analysis of residuals to establish empirical unidimensionality, (3) assess item fit and hierarchical item calibration, and (4) determine person and item reliability as well as separation indices to ensure measurement precision and internal consistency. By integrating regulatory competency mandates with expanded theoretical domains into a Rasch-calibrated measurement framework, this study advances both the conceptual coherence and psychometric robustness of professional competency assessment in school counselors.

## Methods

### Study design

This study employed a quantitative cross-sectional instrumental design aimed at developing and validating a multidimensional instrument for measuring professional competency in school counselors. The research followed a structured process of construct specification, item development, expert validation, and psychometric evaluation using the Rasch Measurement Model. The design was intended to ensure conceptual rigor, measurement precision, and reproducibility.

### Instrument development

The School Counselor Professional Competency Instrument was developed through a structured construct specification process grounded in regulatory, theoretical, and empirical foundations, which is available as extended data ( Khairun, 2026). The initial conceptual framework was derived from the Indonesian Ministry of Education and Culture Regulation No. 111/2014 concerning guidance and counseling in formal education, which specifies four core professional functions: assessment, development of guidance and counseling program, implementation of guidance and counseling services, and evaluation guidance and counseling program.

To strengthen the theoretical comprehensiveness of the construct, the framework was expanded to incorporate three additional domains identified in contemporary professional competency literature: supervision of pre-service counselor training, continuous professional development, and collaborative engagement in co-curricular programs. The inclusion of these domains reflects a broader conceptualization of professional competency that extends beyond technical program delivery to encompass sustainability of the profession, ethical responsibility, and systemic integration within educational settings.

Based on this integrative framework, an item pool was generated to represent seven domains:

a. Assessment of student potential, characteristics, and environment
b. Development of guidance and counseling program
c. Implementation of guidance and counseling services
d. Evaluation of guidance and counseling program
e. Supervision of pre-service counselor training
f. Continuous professional development
g. Collaborative engagement in co-curricular programs

The final instrument consisted of 71 favorable items rated using a five-point Likert scale. The rating scale was designed to capture the behavioral frequency of professional practices in authentic school contexts.

### Content validation

The content validation of the School Counselor Professional Competency Instrument was conducted through a rigorous expert judgment process involving a panel of three senior scholars. The committee comprised experts with specialized proficiency in psychometrics, guidance and counseling supervision, and professional development. This multidisciplinary panel ensured that the instrument attained high conceptual alignment with both regulatory standards and contemporary theoretical advancements in the counseling field.

During the refinement phase, initial item formulations underwent systematic revision based on critical reviewer feedback. The experts recommended replacing ambiguous terms with standardized operational verbs to enhance measurability and professional clarity. For instance, items were adjusted to integrate managerial elements such as designing and reporting and to clarify referral sources, ensuring that the measured competencies reflect a complete range of professional accountability and systematic clinical practice.

Following the expert consensus, a comprehensive readability test was administered to a select group of students possessing demographic and academic characteristics identical to the target population. This stage verified the linguistic structure and sociocultural nuances of the instrument, ensuring it was easily comprehensible to the end-users. Gathering direct feedback allowed researchers to confirm that the items were free from interpretative bias and remained ecologically valid within the Indonesian higher education context.

The final validation strategy employed a robust dual-layered approach that combined theoretical scrutiny from subject-matter experts with student-centered linguistic feedback. This systematic process guaranteed that the instrument was fully optimized for large-scale data collection and subsequent psychometric calibration. Consequently, the validated scale provides a methodologically sound foundation for evaluating professional competency using the Rasch Measurement Model.

### Readability and pilot testing

Following the attainment of expert consensus on item validity, a comprehensive readability assessment was conducted with a group of participants sharing demographic and academic profiles identical to the target subjects. This phase served as a critical bridge between theoretical validation and practical application, ensuring conceptual alignment between the population and the research sample. The primary objective of this assessment was to verify that the linguistic structure and sociocultural nuances of the instrument were readily comprehensible to the end-users, thereby mitigating interpretive bias and establishing ecological validity within the Indonesian educational context.

The readability results indicated high overall comprehension rates (ranging from 3 to 5), yet prompted strategic refinements to enhance clarity and measurement precision. Based on counselor feedback, technical jargon and specific Indonesian acronyms, such as “ITP/ATP,” were supplemented with brief descriptors to prevent data measurement errors, while descriptions of complex administrative tasks were simplified to improve readability and reduce respondent fatigue. Furthermore, items were updated to encompass a broader “school authorities” network, strengthening the instrument’s ecological validity by accurately reflecting the hierarchical referral flow in schools. This dual-layered validation process ensured the instrument was fully optimized for large-scale administration and subsequent Rasch Model analysis.

### Participants and data collection

Data collection for this instrumental study officially commenced on 2 February 2025, following the renewal of ethical approval and within its validity period. The recruitment process targeted 93 practicing school counselors (N = 93) to ensure the ecological validity of the instrument calibration. Demographic analysis of these participants revealed an age range of 24 to 59 years, with a mean age of 34.45 years (SD = 9.40). This demographic diversity provides a robust basis for evaluating professional competency across various career stages within the school counseling profession ( Table 1). Participation was voluntary and anonymous ( Khairun, 2026). Participants were informed about the purpose of the study and the anonymous use of data for research purposes. No personally identifiable information was collected. Responses were coded numerically and prepared in spreadsheet format for Rasch analysis.

**Table 1.** Demographic profile of the participants (N = 93).

Characteristics	Category	Frequency (n)	Percentage (%)
Gender	Female	76	81.7%
	Male	17	18.3%
Education Background	Bachelor of Guidance and Counseling	65	69.9%
	Master of Guidance and Counseling	18	19.4%
	Bachelor of Psychology	5	5.4%
	Others	5	5.4%
Age (Years)	**Range: 24–59**	**Mean: 34.45**	**SD: 9.40**

To minimize potential bias, standardized written instructions were provided to all participants, a uniform five-category response format was used, and anonymous participation was maintained to reduce social desirability effects. Post-hoc diagnostic analysis was conducted to detect potential misfitting responses.

### Rasch measurement analysis

Psychometric evaluation was conducted using the Rasch Rating Scale Model implemented in Winsteps version 5.4.0. The Rasch model was selected because it transforms ordinal Likert responses into interval-level measures, estimates item and person parameters on a common logit scale, and provides rigorous evaluation of measurement properties.

Given the uniform five-category response structure across all items, the Rating Scale Model (Andrich model) was applied. The analysis estimated item difficulty parameters, person ability parameters, item and person fit statistics, reliability indices, separation indices, and Principal Components Analysis of Residuals (PCAR). All 71 items were retained for analysis.

Item fit was evaluated using Infit and Outfit Mean Square statistics. Acceptable fit was defined as MNSQ values between 0.50 and 1.50 and standardized fit statistics within ±2.0. Measurement precision was examined through person reliability, item reliability, person separation index, and item separation index. Unidimensionality was assessed using Principal Components Analysis of Residuals. The raw variance explained by measures was 53.4%, and the unexplained variance in the first contrast was 6.7%, indicating the presence of a dominant latent construct despite the multidomain conceptual structure.

Category functioning was evaluated through category frequency distribution, average measures across categories, and threshold ordering. The five-category Likert scale demonstrated ordered thresholds, indicating appropriate category progression.

## Results

### Descriptive overview of the instrument structure

The instrument consisted of 71 favorable items distributed across seven theoretically defined domains of professional competency in school counselors, as detailed in the instrument blueprint ( Table 2). The final version was refined through a panel of three experts whose profiles are detailed in the Rater Committee ( Table 3). This process involved significant linguistic and conceptual adjustments, which are documented in the Original and Revised Versions ( Table 4) and the Matrix of Counselor Feedback and Linguistic Refinements ( Table 5). Participants responded to the items using a five-category Likert scale, with the response options and their corresponding statistical interpretations defined in the Scoring Guidelines ( Table 6).

**Table 2.** Instrument blueprint of the multidimensional school counselor professional competency instrument.

No	Domain	Indicator	Item	Total
1.	Assessment of Student Potential, Characteristics, and Environment	a. Developing non-test instruments	1	1
		b. Analyzing test and non-test data	2	1
		c. Utilizing Developmental Task Inventory (DTI/ITP)	3	1
		d. Developing cumulative record formats	4	1
		e. Interpreting and explaining psychological test results	5	1
		f. Utilizing student data for school program development	6	1
		g. Utilizing student data for placement	7	1
		h. Utilizing student data for career guidance	8	1
		i. Assisting teachers through student data utilization	9	1
		j. Providing individual and group interventions	10	1
2.	Development of Guidance and Counseling Program	a. Designing school-wide guidance and counseling programs	11, 12	2
		b. Developing operational action plans	13, 14, 15	3
3.	Implementation of Guidance and Counseling Services	a. Delivering core/basic services	16, 17, 18	3
		b. Delivering responsive services	19, 20, 21, 22, 23, 24, 25, 26, 27, 28, 29, 30, 31, 32, 33, 34	16
		c. Utilizing media and technology	35, 36	2
		d. Establishing partnerships	37, 38	2
		e. Participatory duties	39, 40	2
4.	Evaluation of Guidance and Counseling Programs	a. Conducting process and outcome evaluations	41, 42	2
		b. Executing program accountability	43	1
5.	Supervision of Pre-service Counselor Training	a. Implementing supervision programs	44, 45, 46, 47, 48, 49	6
		b. Evaluation and follow-up	50, 51, 52	3
6.	Continous Professional Development	a. Professional community involvement	53, 54, 55	3
		b. Advanced studies	56, 57	2
		c. Professional training and workshops	58, 59, 60	3
		d. Utilizing scientific and public forums	61, 62	2
		e. Research and scholarly works	63, 64, 65	3
7.	Collaborative Engagement in Co-curricular Programs	a. Program planning and developmen	66, 67	2
		b. Mentoring personal development	68, 69	2
		c. Character and value development	70, 71	2
		**Total**		**71**

**Table 3.** Profiles and expertise of expert committee for content validation.

No	Education	Gender	Age and work experience	Expertise	Affiliation
1	**Prof., Dr., M.Pd.**	Male	60/35	Proficiency in psychometrics	Universitas Pendidikan Indonesia
2	**Dr., M.Pd.**	Male	37/11	Expertise in guidance and counselling supervision	Universitas Pendidikan Indonesia
3	**Dr., M.Pd.**	Female	54/9	Professional development in guidance and counseling	Universitas Pendidikan Indonesia

**Table 4.** Original and revised versions of school counselor professional competency instrument.

Item no.	Domain/indicator	Initial item formulation	Expert reviewer feedback	Refined item	Refinement rationale
1	Assessment of student potential, characteristics, and environment/Developing non-test instruments.	Conducting tests and non-tests to identify student problems at school.	The word “conducting” is too general.	Administering psychological tests and developing non-test instruments to collect data on individual student potential, characteristics, and their environment.	Integrating non-test instrument development and environmental factors to ensure assessment competence covers more systemic and contextual data collection.
15	Development of guidance and counseling program/Developing operational action plans	Informing school members about the guidance and counseling activity schedule so they know when it takes place.	The phrasing remains too informative and fails to demonstrate specific professional managerial actions.	Promoting and coordinating the implementation schedule of guidance and counseling program.	Integrating advocacy and systematic organization aspects through the use of more standardized and measurable operational verbs.
21	Implementation of guidance and counseling services/Delivering responsive services	Conducting home visits to provide assistance to students in need.	Your initial phrasing tends to focus only on technical implementation without demonstrating a complete range of professional accountability.	Designing, implementing, and reporting on home visits to understand or assist specific student cases.	Integrating managerial elements (designing and reporting) into operational actions to ensure the measured competence covers systematic and accountable practice standards.
23	Implementation of guidance and counseling services/Delivering responsive services	Assisting in handling students with problems based on reports from school authorities or parents.	The initial phrasing is too general and does not describe systematic clinical or pedagogical work stages.	Receiving and analysing referrals from teachers or parents and providing appropriate interventions.	Operationalizing performance verbs and clarifying referral sources so that the measured competence can be evaluated more objectively and structurally.
51	Supervision of pre-service counselor training/Evaluation and follow-up	Checking guidance and counseling program implementation reports prepared by staff.	The item phrasing needs to be more operational and demonstrate the supervisor’s active role in the document preparation guidance process.	Guiding the supervisee in designing implementation reports for the guidance and counseling programs they conducted.	Increasing competency accuracy from mere result-checking to strategic guidance actions during the report design stage.
65	Continous professional development/Research and scholarly works	Capable of independently conducting action research in guidance and counseling.	This phrasing is very comprehensive in describing the workflow, but is technically double-barreled as it combines four different activities into one statement.	Developing, implementing, and reporting on Action Research in Counseling and presenting findings in scientific forums.	Delineating behavioral indicators specifically from planning to dissemination to ensure the measured competency standards become more concrete and observable.

**Table 5.** Matrix of counselor feedback and linguistic refinements (readability phase).

Item no	Reader ID	Comprehension Rate (1–5)	Initial counselor feedback & comprehension notes	Refinement action/observation	Ecological & face validity impact
1	R1	5 (Very Clear)	The phrase “administering psychological tests” is highly appropriate as it distinguishes our professional role from general teaching staff.	Retained the technical terminology to maintain professional standards	Strengthens the face validity of the instrument as a specialized professional tool.
4	R2	4 (Clear)	“Cumulative data formats” is clear, but some younger counselors might confuse it with digital databases versus physical folders.	Added a note to include both digital and physical formats during the instrument briefing.	Ensures the item is contextually relevant to schools with varying levels of digitalization.
3	R3	3 (Neutral)	Using specific acronyms like “ITP/ATP” (Inventory of Developmental Tasks) might be confusing for those not trained in these specific Indonesian tools.	Decided to keep the acronyms but provided brief descriptors in parentheses for clarity.	Increases linguistic precision and prevents data measurement errors due to jargon.
15	R4	5 (Very Clear)	“Promoting and coordinating” perfectly captures the proactive nature of modern BK management compared to just “informing.”	Phrasing finalized; no further linguistic adjustment needed.	Reflects the transition from a passive service to an active leadership role in schools.
21	R5	3 (Neutral)	The combination of “designing, implementing, and reporting” home visits in one sentence feels wordy and implies a heavy administrative load.	Simplified the sentence structure while maintaining the three-stage accountability process.	Improves readability and prevents “respondent fatigue” when answering long items.
23	R6	4 (Clear)	Suggests that “referral cases” should explicitly include those from the school principal, not just teachers and parents.	Updated the item to include “school authorities” to encompass a broader referral network.	Enhances ecological validity by reflecting the actual hierarchical referral flow in schools
8	R7	5 (Very Clear)	Using student data for “career guidance” is a very high-frequency task that resonates well with high school counsellors.	Retained as a “high-resonance” item for the career counseling indicator.	Solidifies the contextual validity of the instrument for secondary education settings.
5	R8	4 (Clear)	“Interpreting data to relevant parties” raised a question about confidentiality; who are the “relevant parties”?	Refined the wording to “authorized stakeholders” to imply adherence to the code of ethics.	Safeguards professional ethics within the competency measurement framework.
6	R9	5 (Very Clear)	Linking student data to “school program development” shows that BK is not an isolated island but part of the school system.	No adjustments needed; the operational verb is well-received.	Demonstrates the systemic impact of BK services on the broader educational environment.
10	R10	3 (Neutral)	The term “assistance” (bantuan) was felt to be too “charity-based” rather than “empowerment-based.”	Changed “providing assistance” to “facilitating interventions” to reflect a more modern pedagogical approach.	Shifts the perspective from reactive help to proactive, professional psychological intervention.

**Table 6.** Scoring guidelines for school counselor professional competency instrument.

Response options	Favorable	Statistical interpretation
Always	5	Highly Competent
Often	4	Competent
Sometimes	3	Moderately Competent
Rarely	2	Partially Competent
Never	1	Incompetent/Not yet Competent

### Overall model fit and measurement summary

The Rasch calibration produced a well-targeted measurement structure with item difficulty centered at 0.00 logits (SD = 0.76). Item fit statistics showed a mean Infit MNSQ of 0.98 (SD = 0.31) and mean Outfit MNSQ of 1.05 (SD = 0.45), indicating overall conformity to model expectations. As shown in Figure 1, most items fell within acceptable fit criteria (0.50–1.50 MNSQ). Although several items showed elevated ZSTD values due to sample sensitivity, their MNSQ values remained within tolerable ranges, supporting retention of all 71 items.

**Figure 1.**
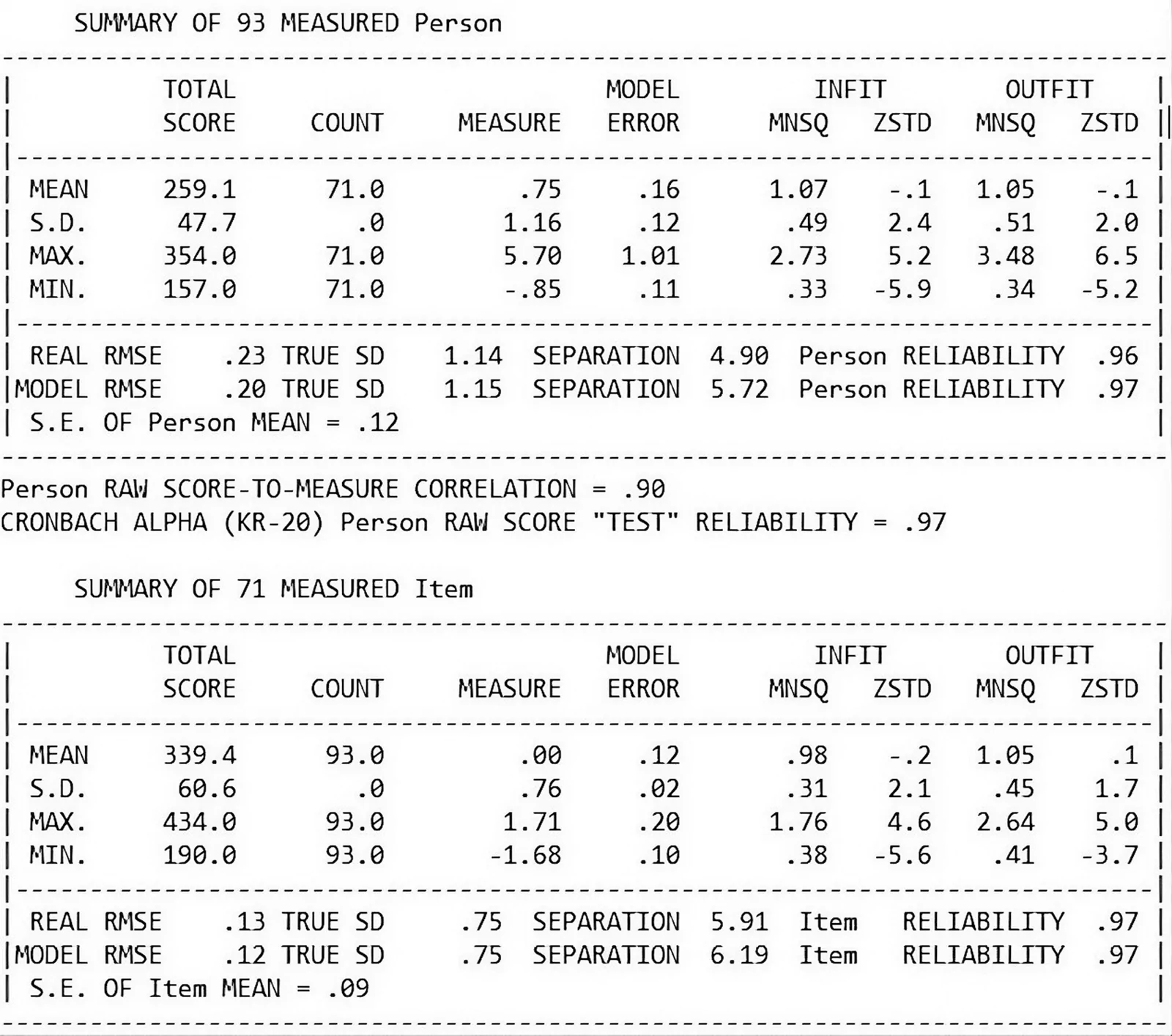
Rasch reliability and measurement summary of the instrument.

This figure presents the overall Rasch calibration statistics, including mean measures, standard deviations, and reliability indices.

### Unidimensionality evidence

Unidimensionality was evaluated using Principal Components Analysis of Residuals (PCAR), with results presented in Table 7.

**Table 7.** Construct validity of school counselor professional competency instrument based on Rasch unidimensionality analysis.

UNDIMENSIONALITY				
Table of STANDARDIZED RESIDUAL				
variance (in Eigenvalue units)				
	Empirical			Model
*Total raw variance in observations*	152.5	100.0%		100.0%
*Raw variance explained by measures*	81.5	53.4%		53.5%
*Raw variance explained by persons*	31.1	20.4%		20.5%
*Raw Variance explained by items*	50.3	33.0%		33.1%
*Raw unexplained variance (total)*	71.0	46.6%	100.0%	46.5%
*Unexplned variance in 1st contrast*	10.3	6.7%	14.5%	
*Unexplned variance in 2nd contrast*	9.0	5.9%	12.6%	
*Unexplned variance in 3rd contrast*	4.5	2.9%	6.3%	
*Unexplned variance in 4th contrast*	3.4	2.3%	4.8%	
*Unexplned variance in 5th contrast*	3.2	2.1%	4.4%	

Key indicators were:

a. Raw variance explained by measures = 53.4%
b. Unexplained variance in first contrast = 6.7%

The variance explained substantially exceeded the 20% minimum criterion for a dominant dimension, while the first contrast remained well below the 15% threshold. These findings support the presence of a strong primary latent construct underlying the instrument despite its seven-domain theoretical structure. The seven domains therefore function as substantively distinct but statistically integrated components of a unified construct: professional competency in school counselors.

### Item difficulty distribution across domains

Item difficulty ranged from −1.68 logits to +1.71 logits, as documented in the Distribution of Item Difficulty Levels ( Table 8). The most difficult items (e.g., items 55, 64 and 65) were primarily located in supervision of pre-service counselor training, research activities, professional leadership roles. Conversely, the easiest items (e.g., items 8, 18, and 23) were concentrated in basic assessment utilization, routine implementation of counseling services, and administrative documentation tasks.

**Table 8.** Distribution of item difficulty levels based on Rasch measurement analysis.

*Entry number*	*Measure*	*Infit*		*Outfit*		*Pt-Me Corr, *	
		*MNSQ*	*ZSTD*	*MNSQ*	*ZSTD*		Keterangan
55	1,71	1,76	3,7	2,62	5	0,51	Very Difficult
64	1,67	1,41	2,2	1,52	2,1	0,62	Sangat Sulit
65	1,39	1,61	3,5	1,66	2,9	0,57	Sangat Sulit
63	1,36	1,29	1,8	1,18	0,9	0,64	Sangat Sulit
52	1,02	1,57	3,7	1,32	1,8	0,61	Sangat Sulit
28	0,97	0,97	-0,2	1,09	0,6	0,63	Sangat Sulit
48	0,9	1,08	0,6	1,09	0,6	0,66	Sangat Sulit
51	0,87	1,17	1,3	1,03	0,2	0,66	Sangat Sulit
57	0,87	1,59	3,9	1,77	3,8	0,5	Sangat Sulit
49	0,84	1,22	1,6	1,05	0,4	0,63	Sangat Sulit
59	0,83	1,08	0,6	1,11	0,7	0,63	Sangat Sulit
47	0,82	1,46	3,1	1,25	1,4	0,61	Sangat Sulit
44	0,78	1,37	2,6	1,18	1,1	0,59	Sangat Sulit
62	0,75	0,79	−1,7	0,81	−1,1	0,66	Difficult
66	0,74	0,87	−1	0,81	−1,1	0,64	Sulit
45	0,71	1,46	3,2	1,25	1,4	0,58	Sulit
58	0,7	0,82	−1,4	0,85	−0,9	0,65	Sulit
53	0,64	1,31	2,2	1,33	1,8	0,59	Sulit
61	0,61	0,85	−1,2	0,8	−1,2	0,65	Sulit
50	0,54	0,97	−0,2	0,86	−0,8	0,62	Sulit
46	0,52	1,38	2,6	1,21	1,2	0,57	Sulit
67	0,46	0,79	−1,6	0,68	−1,9	0,65	Sulit
68	0,42	0,91	−0,6	0,78	−1,3	0,6	Sulit
69	0,39	1,02	0,2	0,88	−0,6	0,59	Sulit
70	0,38	1,02	0,2	0,9	−0,5	0,57	Sulit
3	0,36	1,75	4,6	1,9	3,9	0,35	Sulit
71	0,36	0,92	−0,5	0,77	−1,3	0,61	Sulit
24	0,32	0,84	−1,2	0,97	−0,1	0,54	Sulit
14	0,25	0,42	−5,4	0,64	−2	0,63	Sulit
41	0,15	0,63	−2,9	0,61	−2,2	0,62	Sulit
40	0,1	0,92	−0,5	0,9	−0,4	0,58	Sulit
43	0,08	0,38	−5,6	0,41	−3,7	0,69	Sulit
42	0,06	0,73	−2	0,65	−1,9	0,61	Sulit
17	0,04	0,61	−3	0,93	−0,2	0,55	Sulit
38	0,02	0,45	−4,6	0,47	−3,1	0,67	Sulit
29	0,01	0,78	−1,5	0,71	−1,5	0,59	Sulit
60	−0,12	0,97	−0,1	0,93	−0,3	0,45	Easy
32	−0,17	1,15	0,9	0,99	0	0,48	Mudah
33	−0,19	0,82	−1,1	0,79	−0,9	0,55	Mudah
13	−0,21	0,72	−1,8	0,69	−1,5	0,56	Mudah
54	−0,24	0,84	−1	0,76	−1	0,55	Mudah
56	−0,28	1,55	2,8	1,87	3	0,37	Mudah
15	−0,3	0,71	−1,9	0,73	−1,2	0,5	Mudah
5	−0,31	0,87	−0,7	0,99	0	0,49	Mudah
12	−0,32	0,68	−2,1	0,69	−1,4	0,51	Mudah
22	−0,32	0,7	−1,9	0,7	−1,3	0,54	Mudah
7	−0,34	1,59	3	1,47	1,8	0,36	Mudah
30	−0,35	0,81	−1,1	0,72	−1,2	0,53	Mudah
36	−0,37	0,91	−0,5	0,84	−0,6	0,49	Mudah
39	−0,44	0,58	−2,7	0,61	−1,7	0,55	Mudah
34	−0,46	0,87	−0,7	0,85	−0,5	0,46	Mudah
1	−0,49	1,03	0,2	1,19	0,8	0,46	Mudah
2	−0,49	0,81	−1	2,64	4,6	0,44	Mudah
9	−0,52	0,93	−0,3	1,93	2,9	0,33	Mudah
10	−0,54	0,75	−1,4	1,79	2,6	0,42	Mudah
11	−0,59	0,67	−1,9	0,61	−1,6	0,5	Mudah
31	−0,66	0,89	−0,6	0,79	−0,7	0,46	Mudah
4	−0,72	1,08	0,4	1,54	1,8	0,26	Mudah
37	−0,76	0,84	−0,8	0,78	−0,8	0,46	Mudah
35	−0,78	1,07	0,4	1,25	0,9	0,33	Very Easy
16	−0,82	0,69	−1,7	0,61	−1,5	0,53	Sangat Mudah
6	−0,9	1,06	0,4	1,55	1,7	0,32	Sangat Mudah
27	−0,93	0,73	−1,4	0,67	−1,1	0,45	Sangat Mudah
25	−0,95	0,93	−0,3	0,88	−0,3	0,43	Sangat Mudah
20	−1,07	1,04	0,3	1,12	0,5	0,37	Sangat Mudah
26	−1,07	0,64	−1,9	0,55	−1,6	0,51	Sangat Mudah
21	−1,09	0,77	−1,1	0,79	−0,6	0,41	Sangat Mudah
19	−1,31	0,92	−0,3	0,94	−0,1	0,33	Sangat Mudah
23	−1,37	0,81	−0,9	0,78	−0,6	0,38	Sangat Mudah
18	−1,43	1,19	0,9	1,45	1,3	0,25	Sangat Mudah
8	−1,68	0,8	−0,9	1,2	0,6	0,29	Sangat Mudah
MEAN	0	0,98	−0,2	1,05	0,1		
S,D,	0,76	0,31	2,1	0,45	1,7		

This distribution indicates that advanced professional engagement (e.g., research publication, structured supervision, leadership in professional organizations) represents higher levels of the latent trait, whereas operational service delivery reflects foundational competency. The item hierarchy therefore mirrors developmental progression within the profession.

### Wright map interpretation

The Wright Map ( Figure 2) presents the joint distribution of person ability and item difficulty on the same logit scale.

**Figure 2.**
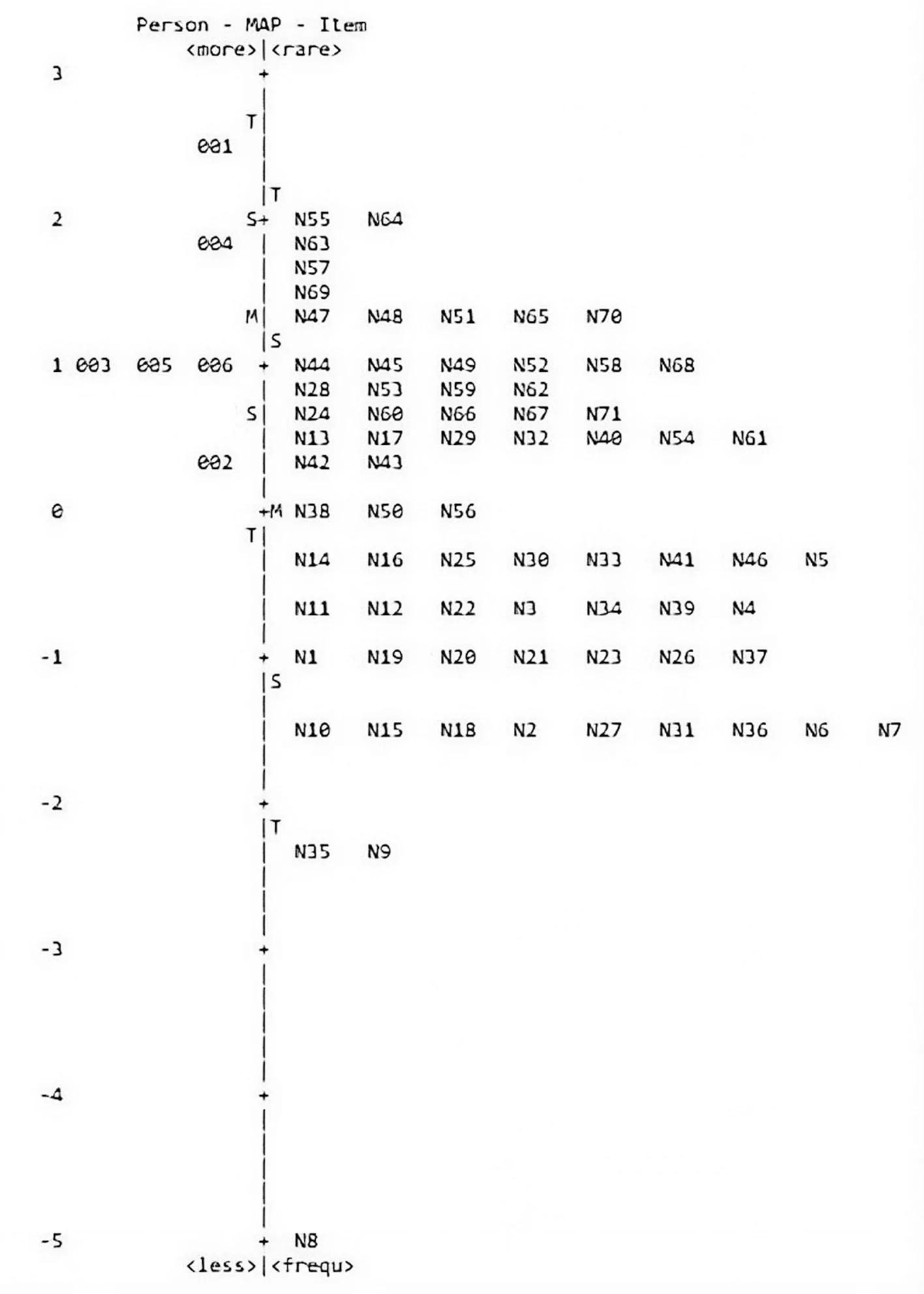
Wright map of school counselor professional competency.

Key observations:

a. Person measures were generally well-targeted to item difficulty range.
b. The mean person ability was slightly higher than item mean (0 logits), indicating that respondents overall demonstrated moderate-to-high professional competence.
c. Items representing Domains 5, 6, and 7 were clustered at higher logit levels, confirming that supervision, research engagement, and systemic collaboration function as advanced competence indicators.
d. Items from Domains 1–3 were more evenly distributed across mid-level logits, reflecting core operational practice.

The Wright Map demonstrates adequate coverage of the latent continuum without major ceiling or floor effects.

This figure illustrates the distribution of person ability and item difficulty on the same logit scale. Higher logit values represent more advanced competencies, while lower logit values reflect foundational counseling practices.

### Rating scale functioning

Category functioning diagnostics are displayed in Figure 3 and Figure 4. Analysis showed:

a. All five categories were utilized.
b. Average measures advanced monotonically across categories.
c. Thresholds were properly ordered.

**Figure 3.**
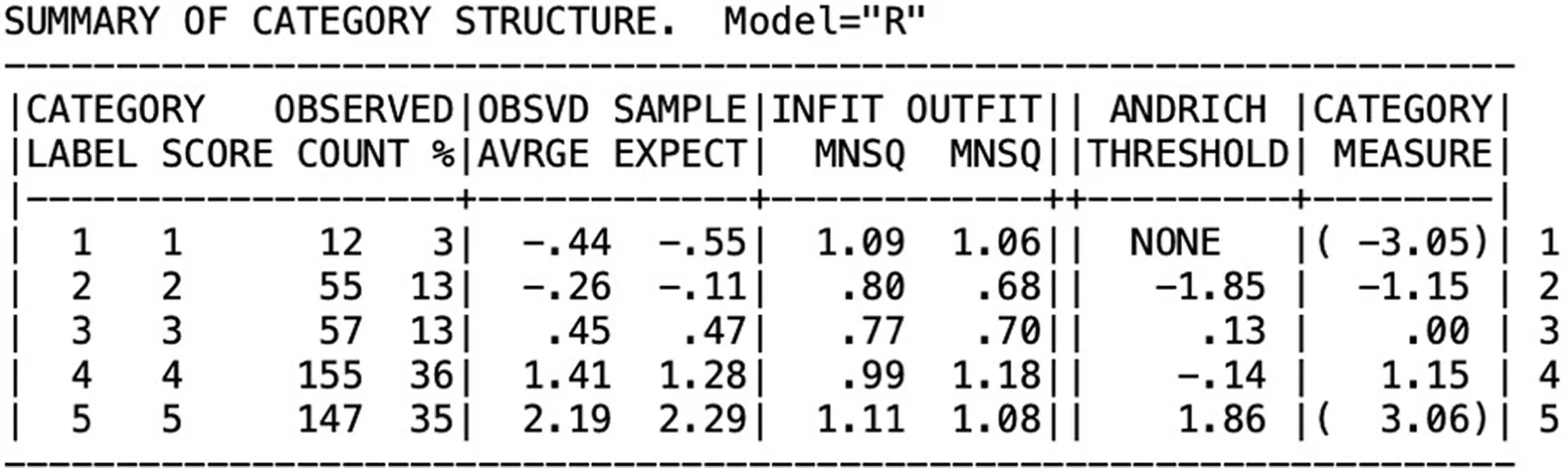
Category functioning and rating scale diagnostics.

**Figure 4.**
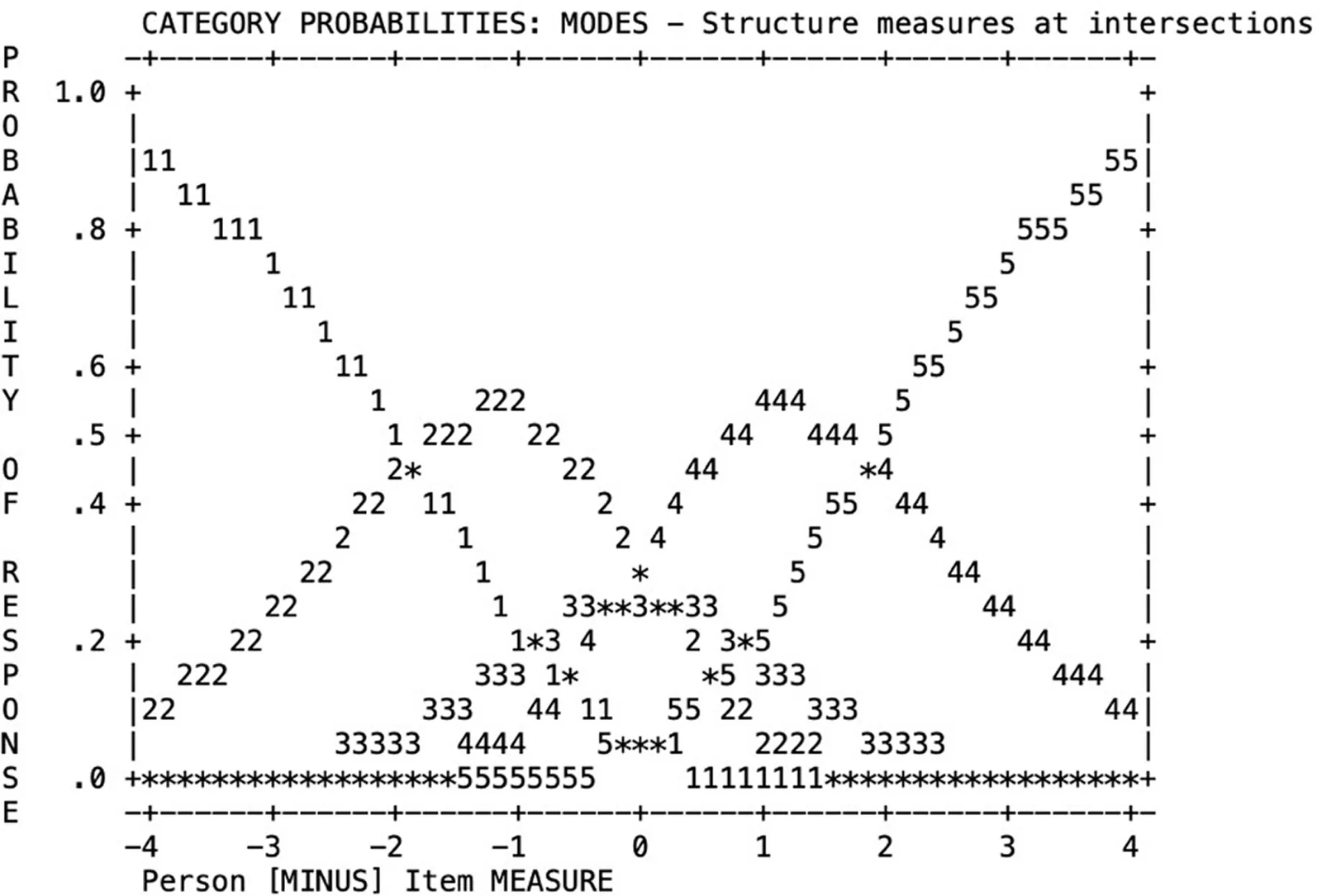
Probability curves for rating scale categories.

These findings indicate that respondents meaningfully differentiated between response categories, and the five-point Likert structure functioned as intended. The scale progression reflects increasing behavioral frequency of authentic professional practice.

This figure shows that average measures increase monotonically across categories and that thresholds are properly ordered, indicating appropriate functioning of the five-point Likert scale.

This plot displays distinct peaks for each response category, indicating effective differentiation among the five Likert scale options without significant overlap.

### Reliability and separation indices

Reliability results are summarized in Figure 1. The analysis demonstrated:

a. High item reliability (indicating stable item hierarchy)
b. Adequate person reliability (indicating consistent ordering of respondents)
c. Satisfactory separation indices for both persons and items

The instrument was therefore capable of distinguishing multiple strata of professional competency among school counselors.

### Construct validity synthesis

Taken together, strong variance explained (53.4%), low first contrast (6.7%), acceptable item fit, ordered rating scale structure, adequate reliability and separation, and a theoretically coherent item hierarchy across seven domains provide converging evidence that the instrument demonstrates:

a. Structural validity
b. Internal construct coherence
c. Measurement precision
d. Functional multidimensional integration within a dominant latent construct

The seven domains operate as theoretically meaningful facets while remaining statistically unified within a single Rasch-calibrated measurement framework.

## Discussion

This study advances the measurement of professional competency in school counselors by integrating regulatory mandates and contemporary theoretical expansions into a Rasch-calibrated framework. Although the instrument was theoretically structured across seven domains, Rasch principal component analysis of residuals demonstrated a dominant latent construct, with variance explained exceeding recommended criteria and first-contrast residual variance well below critical thresholds ( Boone et al., 2013; Sumintono & Widhiarso, 2015). These findings provide empirical support for the argument that professional competency, while conceptually multidimensional, operates as an integrated and hierarchically organized capability in applied educational settings ( Bernard & Goodyear, 2014; Kaslow, 2004).

The hierarchical ordering of item difficulty offers substantive theoretical insight. Foundational practices such as routine assessment and implementation of counseling services were positioned at lower logit levels, whereas supervision of trainees, engagement in research and scholarly production, and leadership within professional communities were located at higher levels. This ordering aligns with competency-based supervision frameworks that conceptualize professional growth as developmental progression from operational proficiency toward reflective, supervisory, and systemic expertise ( Bernard & Goodyear, 2019; Borders, 2014, 2019). It also reinforces the view that sustainable professional competency requires more than technical service delivery, encompassing mentorship, reflective practice, and institutional collaboration ( Luke & Bernard, 2006; Stoll et al., 2006).

Methodologically, the study contributes by moving beyond classical test theory approaches that dominate competency measurement research. While classical procedures remain useful for internal consistency estimation, they do not ensure invariant item calibration or interval-level scaling ( Bond, 2015; Boone et al., 2013; Sumintono & Widhiarso, 2015). By employing the Rasch Rating Scale Model, this study establishes item and person parameters on a shared metric, enabling objective measurement independent of sample-specific score distributions. The ordered category thresholds and satisfactory separation indices further confirm that the instrument meaningfully differentiates multiple strata of professional competency, strengthening interpretability for supervision and policy applications ( Bond, 2015; Boone et al., 2013).

The inclusion of supervision of trainees, continuous professional development, and collaborative engagement extends traditional regulatory frameworks that often prioritize programmatic implementation alone. Contemporary scholarship increasingly emphasizes that professional competency must incorporate supervisory alliance, reflective capacity, and systemic integration within educational ecosystems ( Bernard & Goodyear, 2019; Borders, 2014; Luke & Bernard, 2006). By empirically demonstrating that these domains function as higher-order indicators within a unified competency continuum, the present study provides evidence for a more holistic and sustainability-oriented model of counselor professionalism ( Kaslow, 2004; Stoll et al., 2006).

The novelty of this work lies in three interrelated contributions. First, it operationalizes a regulatory-theoretical synthesis into a coherent measurement architecture rather than assessing fragmented skill domains. Second, it empirically demonstrates hierarchical integration of advanced professional roles within a single latent construct. Third, it applies Rasch modeling to establish interval-level measurement and hierarchical calibration, thereby strengthening the precision and policy relevance of competency assessment ( Bond, 2015; Boone et al., 2013; Sumintono & Widhiarso, 2015). In doing so, the study responds to calls for more rigorous, competency-based supervision research grounded in robust psychometric methodology ( Bernard & Goodyear, 2019; Borders, 2019).

Several limitations warrant consideration. The cross-sectional design and moderate sample size limit generalizability, although Rasch estimation remains stable within this range ( Sumintono & Widhiarso, 2015). Self-reported frequency measures may introduce response bias, and future research should incorporate multi-source or performance-based assessments. Further analysis of differential item functioning and longitudinal sensitivity would strengthen evidence of invariance and developmental responsiveness ( Bond, 2015; Boone et al., 2013).

Overall, the findings position this instrument as a theoretically integrative and psychometrically rigorous tool for professional supervision, development planning, and policy evaluation within school counseling systems. By demonstrating hierarchical coherence and measurement precision within a unified competence framework, the study contributes both conceptual clarification and methodological advancement to the field of counselor education and supervision ( Bernard & Goodyear, 2019; Borders, 2014; Kaslow, 2004).

## Conclusion

This study developed and Rasch-validated a multidimensional instrument for measuring professional competency in school counselors. The findings demonstrate strong structural validity, coherent hierarchical calibration, appropriate rating scale functioning, and robust reliability and separation indices. Although conceptually organized into seven domains, the instrument operates empirically as a unified latent construct, supporting the interpretation of professional competency as an integrated and hierarchically structured capability rather than a collection of isolated skills.

The calibrated item hierarchy indicates that foundational counseling practices represent core competence, while supervision, scholarly engagement, and systemic collaboration function as advanced indicators of professional maturity. This structure provides a scalable framework for assessing developmental progression within the profession.

Beyond psychometric robustness, the instrument offers practical utility for informing supervision systems, professional development pathways, certification benchmarks, and policy evaluation. With further cross-context validation and longitudinal testing, it has the potential to serve as a standardized reference model for competency-based school counseling systems at both national and international levels.

## Declaration of funding (acknowledgment)

The authors wish to express their highest appreciation and profound gratitude to the Ministry of Finance of the Republic of Indonesia, through the Indonesia Endowment Fund for Education (LPDP), as well as to the Ministry of Higher Education, Science, and Technology of the Republic of Indonesia, through the Center for Higher Education Funding and Assessment (PPAPT) and the Indonesian Education Scholarship (BPI) program, for the financial support, facilitation, and sponsorship provided throughout the publication process of this research.

## Ethics and consent

This study was conducted in accordance with international ethical standards for research involving human subjects. Ethical approval was formally granted by the Research Ethics Committee of Universitas Pendidikan Indonesia (Approval Nos. 9403/UN40.A1.1/TD.07/2024 and 1391/UN40.A1.07/2025). The initial approval was valid from 1 September 2024 to 31 January 2025 and was subsequently renewed for the period from 1 February 2025 to 31 August 2025. Data collection commenced on 2 February 2025, fully within the validity period of the renewed ethical approval. Participation was voluntary and anonymous, and all data were treated confidentially and used solely for research purposes.

## Data Availability

OSF: Measuring Professional Competency in School Counselors: Development and Rasch Validation of a Multidimensional Instrument. https://doi.org/10.17605/OSF.IO/CJBXR ( Khairun, 2026). This project contains the following underlying data:
• A Data Recapitulation of 93 School Counselors Who Have Completed School Counselor Professional Competency Instrument.xlsx: This file contains the anonymized raw response data from 93 practicing school counselors in Indonesia. Data are available under the terms of the Creative Commons Zero “No rights reserved” data waiver (CC0 1.0 Public domain dedication). OSF: Measuring Professional Competency in School Counselors: Development and Rasch Validation of a Multidimensional Instrument. https://doi.org/10.17605/OSF.IO/CJBXR ( Khairun, 2026). This project contains the following extended data:
• School Counselor Professional Competency Instrument.pdf: The full version of the validated 71-item Professional Competency Scale for School Counselors. • Ethical approval certificate No. 9403 (valid from 1 September 2024 to 31 January 2025). • Ethical approval certificate No. 1391 (valid from 1 February 2025 to 31 August 2025). • README.txt: Documentation providing a detailed overview of the folder structure, data dictionary, and licensing information for the project. Data are available under the terms of the Creative Commons Attribution 4.0 International license (CC-BY 4.0).
