# Supplementary material for: Measuring Professional Competency in School Counselors: Development and Rasch Validation of a Multidimensional Instrument
Source: F1000Res. 2026 Apr 22;15:603. [Version 1] doi: 10.12688/f1000research.178940.1 (PMC13644969; doi:10.12688/f1000research.178940.1)
Supplement: Peer review history

# Reviewer report: Reviewer response for version 1

**DOI:** 10.5256/f1000research.197385.r481647

**Published:** 2026-05-12

**Authors:** Mohamad Awal Lakadjo

**Affiliations:** 1 Universitas Negeri Gorontalo, Gorontalo, Indonesia

Article Summary

 The manuscript *“Measuring Professional Competency in School Counselors: Development and Rasch Validation of a Multidimensional Instrument”* reports the development and initial Rasch validation of a 71-item instrument for assessing school counselors’ professional competency. The instrument covers seven domains: assessment, program development, service implementation, program evaluation, supervision of pre-service counselors, continuous professional development, and collaborative engagement in co-curricular programs. Data were collected from 93 practicing school counselors and analyzed using the Rasch Rating Scale Model, including item/person fit, rating scale functioning, reliability, separation indices, item difficulty hierarchy, Wright Map, and principal component analysis of residuals (PCAR).

 The study addresses an important issue in counselor education and professional supervision. Its main strength lies in integrating Indonesian regulatory standards with broader competency domains and applying Rasch measurement rather than relying only on Classical Test Theory. However, the manuscript should be framed more cautiously as an initial validation study. The evidence is promising but not yet sufficient to support strong claims about robust structural validity, measurement invariance, standardization, or policy-level application.

**Responses to Review Questions**

 1. Is the work clearly and accurately presented and does it cite the current literature?

**Answer: Partly**

 The manuscript is generally clear and follows a standard empirical structure. The rationale for developing a competency instrument is understandable, and the cited literature is relevant to school counseling, supervision, professional development, and Rasch measurement.

 However, the Introduction is repetitive, especially in discussing fragmented competency measures, limitations of Classical Test Theory, and the benefits of Rasch analysis. The construct framing also needs clarification. The instrument is described as multidimensional, but the analysis emphasizes a dominant unidimensional Rasch structure. The authors should state whether the scale is intended to represent seven distinct dimensions, a general professional competency factor, or a hierarchical construct with seven related domains.

 The literature review should also be updated with recent studies on school counselor competency assessment, measurement invariance, Rasch validation, and contemporary validity frameworks.

**Must be addressed:** Clarify the construct model, reduce repetition, refine the novelty claim, and strengthen the literature base with recent and internationally relevant sources.

 2. Is the study design appropriate and is the work technically sound?

**Answer: Partly**

 A cross-sectional instrumental design is appropriate for initial scale development. The Rasch Rating Scale Model is also suitable because all items use the same five-point response structure.

 The main limitation is methodological strength. The sample size of 93 respondents for 71 items is modest for a newly developed instrument covering seven competency domains. Although Rasch analysis can be conducted with relatively small samples, the manuscript makes broad claims about item hierarchy, structural validity, and practical use in supervision and policy. These claims require either stronger empirical support or more cautious wording.

 The sampling procedure is insufficiently reported. The manuscript should explain recruitment strategy, participant eligibility, geographic distribution, school level, and institutional context. The exclusive use of self-report data is another limitation because professional competency ratings may be affected by social desirability and self-perception bias.

**Must be addressed:** Provide detailed sampling information, justify sample adequacy for Rasch calibration, acknowledge self-report bias, and moderate claims that exceed the study design.

 3. Are sufficient details of methods and analysis provided to allow replication by others?

**Answer: Partly**

 The manuscript reports key methodological elements, including item number, competency domains, response format, expert review, readability testing, software, Rasch model, fit criteria, reliability indices, separation indices, and PCAR. The availability of source and extended data is a strength.

 However, several procedures remain insufficiently specified. The recruitment process is unclear. The readability testing phase lacks information on participant number, characteristics, selection criteria, and feedback analysis. Content validation is presented qualitatively, but no quantitative evidence is reported, such as Aiken’s V, Content Validity Index, or inter-rater agreement. Data screening procedures are also unclear, including missing data handling, person misfit evaluation, and criteria for retaining all 71 items.

**Must be addressed:** Expand the Methods section to describe recruitment, participant selection, readability testing, content validity procedures, missing data handling, person/item misfit decisions, and item retention criteria.

 4. If applicable, is the statistical analysis and its interpretation appropriate?

**Answer: Partly**

 The selected statistical approach is appropriate. Rasch analysis is suitable for evaluating item difficulty, fit statistics, response category functioning, person-item targeting, reliability, and separation. The reported outputs provide useful preliminary evidence of instrument functioning.

 The main concern is interpretation. The manuscript emphasizes the 53.4% raw variance explained and 6.7% unexplained variance in the first contrast, but it does not adequately discuss the first contrast eigenvalue of 10.3. Given the seven-domain framework, residual dimensionality requires more careful interpretation.

 Item fit also needs stronger justification. Some items show elevated fit statistics or ZSTD values, yet all items are retained. The authors should explain whether these items are theoretically essential, diagnostically acceptable, or in need of revision. In addition, no Differential Item Functioning (DIF) analysis is reported, despite variation in gender, age, and educational background. Therefore, claims related to invariance should be limited.

**Must be addressed:** Reinterpret PCAR results more cautiously, discuss the first contrast eigenvalue, justify retention of potentially misfitting items, conduct DIF analysis where feasible, or clearly state its absence as a limitation.

 5. Are all the source data underlying the results available to ensure full reproducibility?

**Answer: Yes**

 The manuscript states that anonymized raw response data are available through OSF, together with the full instrument, ethical approval documents, and supporting materials. This supports transparency and reproducibility.

 Reproducibility would be improved by adding the Winsteps control file, complete output files, item coding guide, scoring documentation, and a brief explanation linking each reported table and figure to the analysis output.

**Recommended improvement:** Provide complete Rasch analysis files and coding documentation alongside the raw data.

 6. Are the conclusions drawn adequately supported by the results?

**Answer: Partly**

 The results support a cautious conclusion that the instrument shows preliminary evidence of acceptable rating scale functioning, internal consistency, item calibration, and hierarchical item difficulty within the present sample.

 However, the conclusion overstates the evidence. Claims about strong structural validity, robust measurement precision, certification use, policy evaluation, and international applicability are premature without larger and more diverse samples, DIF analysis, external criterion validation, and cross-context replication.

**Must be addressed:** Revise the conclusion to present the study as an initial Rasch validation. Practical applications should be framed as future possibilities requiring further validation.

 Essential Revisions Required for Scientific Soundness

1. Clarify whether the instrument represents seven dimensions, one general factor, or a hierarchical multidimensional construct.
2. Justify the sample size and describe the sampling procedure in detail.
3. Provide quantitative content validity evidence or acknowledge its absence as a limitation.
4. Report readability/pilot testing procedures transparently.
5. Explain item retention decisions, especially for items with elevated fit indices or ZSTD values.
6. Interpret PCAR results more carefully, particularly the first contrast eigenvalue.
7. Conduct DIF analysis where possible or clearly identify its absence as a limitation.
8. Moderate claims about measurement invariance, structural validity, standardization, certification, and policy use.
9. Standardize terminology, spelling, and table labels, including the English translation of difficulty categories in Table 8.
10. Update the literature review with recent work on counselor competency measurement, Rasch validation, measurement invariance, and validity theory.

**Overall Recommendation**

**Major Revision**

 The manuscript addresses a relevant problem and offers a promising initial instrument for measuring school counselors’ professional competency. The integration of regulatory standards, competency theory, and Rasch measurement is valuable. However, the manuscript requires substantial methodological clarification, more precise construct framing, stronger psychometric interpretation, and more cautious conclusions before it can be considered scientifically sound.

Is the work clearly and accurately presented and does it cite the current literature?

Partly

If applicable, is the statistical analysis and its interpretation appropriate?

Partly

Are all the source data underlying the results available to ensure full reproducibility?

Yes

Is the study design appropriate and is the work technically sound?

Partly

Are the conclusions drawn adequately supported by the results?

Partly

Are sufficient details of methods and analysis provided to allow replication by others?

Partly

Reviewer Expertise:

Psychometrics, Rasch Measurement Model, Educational Measurement, Instrument Validation, Guidance and Counseling or School Counseling

I confirm that I have read this submission and believe that I have an appropriate level of expertise to confirm that it is of an acceptable scientific standard, however I have significant reservations, as outlined above.

# Response

**Published:** 2026-08-16

**Authors:** Deasy Yunika Khairun

**Affiliations:** Guidance and Counseling, Universitas Sultan Ageng Tirtayasa, Serang, Banten, Indonesia

We sincerely thank the reviewer for the careful evaluation of our manuscript and for the constructive comments. We greatly appreciate the positive assessment regarding the clarity of the manuscript, the appropriateness of the study design, the methodological rigor, the availability of source data, and the overall scientific quality of the work. We carefully considered all suggestions and revised the manuscript accordingly. All modifications are highlighted using **Track Changes** in the revised manuscript.

**Comment 1**

*The article will be better equipped to clarify its findings and innovations if supplemented with contemporary literature in the introduction and discussion sections. In the introduction, it is essential to cite literature regarding professional competence from both global and Indonesian perspectives.*

**Response**

 Thank you for this valuable suggestion.

 We have substantially revised the **Introduction** by incorporating additional contemporary literature from both international and Indonesian contexts. The revised manuscript now provides a stronger conceptual foundation by integrating recent studies on professional competency in school counseling, Indonesian regulatory standards (Permendikbud No. 111/2014), supervision, professional development, collaborative practice, and contemporary psychometric validation using the Rasch Measurement Model.

 In addition, the **Discussion** has been strengthened by relating our findings to more recent international studies, thereby providing a clearer interpretation of the study's contribution within the broader literature.

**Revision made in:** Introduction and Discussion.

**Comment 2**

*Furthermore, the procedural steps of the Rasch model as an analytical technique should be elaborated upon within the discussion.*

**Response**

 Thank you for this important methodological recommendation.

 In response, we expanded the **Discussion** by providing a more comprehensive explanation of the Rasch validation process adopted in this study. The revised discussion now explains how the sequential analyses—including rating scale functioning, item and person fit statistics, reliability and separation indices, Wright Map interpretation, and Principal Components Analysis of Residuals (PCAR)—collectively provide evidence regarding measurement precision, construct coherence, and hierarchical item calibration.

 This additional explanation clarifies the methodological contribution of applying the Rasch Measurement Model beyond conventional Classical Test Theory approaches.

**Revision made in:** Discussion.

**Comment 3**

*Finally, the conclusion must explicitly present the research findings that constitute the core innovation of this study.*

**Response**

 We appreciate this valuable suggestion.

 The **Conclusion** has been revised to more explicitly highlight the principal findings and the main innovation of the study. The revised conclusion now emphasizes that this study developed a Rasch-calibrated multidimensional professional competency instrument integrating Indonesian regulatory competency standards with broader contemporary theoretical domains. The conclusion also clearly states that the instrument provides preliminary psychometric evidence supporting hierarchical item calibration, structural coherence, and measurement precision, while acknowledging that further validation across broader educational contexts remains necessary.

 This revision clarifies both the scientific contribution and the novelty of the study.

**Revision made in:** Conclusion.

**Overall Response**

 Beyond addressing the specific comments above, we carefully reviewed the entire manuscript to improve conceptual clarity, methodological transparency, and consistency throughout the paper. These revisions strengthen the theoretical foundation, improve the interpretation of the Rasch analysis, and present the study's contribution more explicitly while maintaining appropriate scientific caution.

 Once again, we sincerely thank the reviewer for the thoughtful and constructive feedback. We believe that these revisions have substantially strengthened the quality, clarity, and overall contribution of the manuscript.

# Reviewer report: Reviewer response for version 1

**DOI:** 10.5256/f1000research.197385.r478756

**Published:** 2026-05-06

**Authors:** Sigit Sanyata

**Affiliations:** 1 Universitas Negeri Yogyakarta, Yogyakarta, Special Region of Yogyakarta, Indonesia

The article will be better equipped to clarify its findings and innovations if supplemented with contemporary literature in the introduction and discussion sections. In the introduction, it is essential to cite literature regarding professional competence from both global and Indonesian perspectives. Furthermore, the procedural steps of the Rasch model as an analytical technique should be elaborated upon within the discussion. Finally, the conclusion must explicitly present the research findings that constitute the core innovation of this study. The article will be better equipped to clarify its findings and innovations if supplemented with contemporary literature in the introduction and discussion sections. In the introduction, it is essential to cite literature regarding professional competence from both global and Indonesian perspectives. Furthermore, the procedural steps of the Rasch model as an analytical technique should be elaborated upon within the discussion. Finally, the conclusion must explicitly present the research findings that constitute the core innovation of this study.

Is the work clearly and accurately presented and does it cite the current literature?

Yes

If applicable, is the statistical analysis and its interpretation appropriate?

I cannot comment. A qualified statistician is required.

Are all the source data underlying the results available to ensure full reproducibility?

Yes

Is the study design appropriate and is the work technically sound?

Yes

Are the conclusions drawn adequately supported by the results?

Yes

Are sufficient details of methods and analysis provided to allow replication by others?

Yes

Reviewer Expertise:

My research focuses on counseling topis, specifically Solution-Focused Brief Therapy (SFBT) and gender issues in counseling, with a particular emphasis on Feminist Therapy.

I confirm that I have read this submission and believe that I have an appropriate level of expertise to confirm that it is of an acceptable scientific standard, however I have significant reservations, as outlined above.

# Response

**Published:** 2026-08-12

**Authors:** Deasy Yunika Khairun

**Affiliations:** Guidance and Counseling, Universitas Sultan Ageng Tirtayasa, Serang, Banten, Indonesia

We sincerely thank the reviewer for the careful evaluation of our manuscript and for the constructive and insightful suggestions. We greatly appreciate the reviewer's positive assessment of the study design, methodological rigor, availability of source data, and the overall scientific quality of the manuscript. We have carefully considered every recommendation and have substantially revised the manuscript accordingly. All revisions are visible using **Track Changes** in Version 2. Our point-by-point responses are provided below.

**Comment 1**

*The article will be better equipped to clarify its findings and innovations if supplemented with contemporary literature in the introduction and discussion sections. In the introduction, it is essential to cite literature regarding professional competence from both global and Indonesian perspectives.*

**Response**

 Thank you for this valuable suggestion.

 We have substantially revised the **Introduction** by incorporating additional contemporary literature from both international and Indonesian contexts to strengthen the conceptual foundation of professional competency in school counseling. The revised introduction now better integrates international competency frameworks, Indonesian regulatory standards, and recent empirical studies concerning counselor professionalism, supervision, continuous professional development, and collaborative practice.

 Specifically, we expanded the discussion of:

- contemporary international perspectives on professional competency;
- Indonesian regulatory competency standards (Permendikbud No. 111/2014);
- recent literature concerning supervision, professional development, and collaborative engagement;
- current psychometric literature supporting Rasch-based instrument validation.

These additions provide a stronger theoretical rationale for the development of the proposed multidimensional competency framework.

**Revision made in:** Introduction.

**Comment 2**

*Furthermore, the procedural steps of the Rasch model as an analytical technique should be elaborated upon within the discussion.*

**Response**

 We appreciate this important methodological recommendation.

 In response, we expanded the **Discussion** by adding a dedicated paragraph explaining the sequential Rasch validation procedure adopted in this study. Rather than simply stating that Rasch analysis was used, we now describe the specific analytical stages and their respective purposes, including:

- rating scale functioning,
- item and person fit analysis,
- reliability and separation indices,
- Wright map interpretation,
- Principal Components Analysis of Residuals (PCAR).

The revised manuscript also explains how these complementary analyses collectively provide evidence regarding response category functioning, internal measurement quality, hierarchical item calibration, and dimensional structure, thereby offering a more comprehensive psychometric evaluation than reliance on conventional reliability coefficients alone.

 This additional explanation clarifies the methodological contribution of applying the Rasch Measurement Model beyond traditional Classical Test Theory approaches.

**Revision made in:** Discussion.

**Comment 3**

*Finally, the conclusion must explicitly present the research findings that constitute the core innovation of this study.*

**Response**

 Thank you for this helpful suggestion.

 We revised the **Conclusion** to explicitly state the principal innovation and contribution of the study. The revised conclusion now emphasizes that the major contribution lies in the development of a Rasch-calibrated hierarchical multidimensional competency framework integrating Indonesian regulatory standards with contemporary international perspectives on counselor professionalism.

 The conclusion also highlights that the instrument represents an integrated competency framework rather than a collection of isolated competency domains, while emphasizing that broader validation across educational contexts remains necessary.

 This revision makes the novelty of the study substantially clearer and more directly aligned with the overall findings.

**Revision made in:** Conclusion.

**Comment 4**

*The article will be better equipped to clarify its findings and innovations...*

**Response**

 Beyond addressing each specific recommendation above, we conducted a comprehensive revision throughout the manuscript to improve clarity, conceptual coherence, methodological transparency, and consistency of interpretation.

 The revised version additionally includes:

- strengthened discussion of the multidimensional conceptual framework;
- more balanced interpretation of Rasch validation results;
- clearer distinction between preliminary psychometric evidence and broader validity claims;
- expanded discussion of study limitations and future validation requirements;
- improved consistency of terminology throughout the manuscript.

These revisions collectively improve the clarity, methodological transparency, and overall contribution of the manuscript.

 Once again, we sincerely thank the reviewer for the constructive comments. We believe these revisions have substantially strengthened the manuscript and improved its conceptual clarity, methodological transparency, and scientific contribution.
